# Intravascular Immune Surveillance by CXCR6^+^ NKT Cells Patrolling Liver Sinusoids

**DOI:** 10.1371/journal.pbio.0030113

**Published:** 2005-04-05

**Authors:** Frederic Geissmann, Thomas O Cameron, Stephane Sidobre, Natasha Manlongat, Mitchell Kronenberg, Michael J Briskin, Michael L Dustin, Dan R Littman

**Affiliations:** **1**Molecular Pathogenesis Program, Skirball Institute of Biomolecular MedicineNew York University School of Medicine, New York, New YorkUnited States of America; **2**La Jolla Institute for Allergy and Immunology, San DiegoCaliforniaUnited States of America; **3**Millenium Pharmaceuticals, CambridgeMassachusettsUnited States of America; **4**Howard Hughes Medical Institute, Skirball Institute of Biomolecular MedicineNew York University School of Medicine, New York, New YorkUnited States of America; University of MinnesotaUnited States of America

## Abstract

We examined the in vivo behavior of liver natural killer T cells (NKT cells) by intravital fluorescence microscopic imaging of mice in which a green fluorescent protein cDNA was used to replace the gene encoding the chemokine receptor CXCR6. NKT cells, which account for most CXCR6^+^ cells in liver, were found to crawl within hepatic sinusoids at 10–20 μm/min and to stop upon T cell antigen receptor activation. CXCR6-deficient mice exhibited a selective and severe reduction of CD1d-reactive NKT cells in the liver and decreased susceptibility to T-cell-dependent hepatitis. CXCL16, the cell surface ligand for CXCR6, is expressed on sinusoidal endothelial cells, and CXCR6 deficiency resulted in reduced survival, but not in altered speed or pattern of patrolling of NKT cells. Thus, NKT cells patrol liver sinusoids to provide intravascular immune surveillance, and CXCR6 contributes to liver-based immune responses by regulating their abundance.

## Abstract 2

Intravital fluorescence microscopy shows that natural killer T cells patrol blood vessels in the liver and stop upon activation, demonstrating their role in liver immune surveillance

## Introduction

In both rodents and humans, the liver is the largest solid organ, and performs critical immunological and metabolic functions. The liver receives nutrient-rich blood from the gut via the portal vein and oxygenated blood from the hepatic artery. It processes blood to remove toxins, synthesizes the majority of serum proteins and lipids, stores glycogen, and performs extensive lipid, cholesterol, and vitamin chemistry and storage. The liver is thought to provide a unique environment for lymphocytes, favoring tolerogenic immune system responses, possibly to prevent reactivity to harmless food antigens [1]. However, in response to certain stimuli, acute inflammatory reactions can occur, and result in hepatocyte death (hepatitis) and subsequent regeneration, with progressive fibrosis when stimuli are sustained. Several progressive liver diseases that can lead to liver failure have an autoimmune component [2].

The liver is an important site of visceral infection. The low-pressure circulation and high surface area of contact between blood and parenchymal cells and the high phagocytic capacity of multiple cell types in liver provide pathogens with an easy route of access. The tolerizing environment may additionally contribute to immune avoidance. The World Health Organization estimates that approximately 5% and 3% of the world's population carry hepatitis B and hepatitis C virus, respectively [3]. Many of these cases result in chronic infections that can lead to fatal complications, including hepatocellular carcinoma, cirrhosis, or hemorrhage. Malaria and leishmania also display important liver tropisms [4,5]. Thus, immune surveillance of the liver for pathogens is an important, but poorly understood, process.

The profile of steady-state hepatic immune cells differs markedly from that in secondary lymphoid organs and in other non-lymphoid tissues, with abundant Kupffer cells and natural killer T cells (NKT cells) supplemented with αβ T cells, γδ T cells, natural killer (NK) cells, dendritic cells, and few, if any, B cells. NKT cells, present at trace levels (<1%) in many organs, are highly enriched in the liver, where they represent up to 30% of lymphocytes [6]. These lymphocytes co-express NK receptors (e.g., NK1.1) and T cell antigen receptors (TCRs). The largest subset of NKT cells includes thymus-derived CD1d-reactive cells, most of which express a semi-invariant TCR containing Vα14–Jα18 and Vα24–Jα15 rearrangements in mouse and human, respectively, and a restricted Vβ repertoire [7]. Nearly all of these lymphocytes react with the marine-sponge-derived glycolipid α-galactosyl ceramide (αGalCer) presented by CD1d. The CD1d-specific NKT cells are required for Concanavalin A (ConA)–induced hepatitis [8,9,10], a model of autoimmune hepatitis [11], and are implicated in a variety of other hepatic immunological reactions, including tumor rejection, inhibition of hepatitis B replication, and anti-malarial responses [7].

Lymphocyte trafficking to and within lymphoid organs and also to extra-lymphoid organs such as inflamed gut and skin is governed in large part by the interactions of selectively expressed adhesion receptors and chemokine receptors on the surface of diverse lymphocytes with ligands on the endothelium [12]. This is followed by extravasation of the adherent lymphocytes across the endothelium. This paradigm of lymphocyte homing has emerged mainly from studies of high-velocity vascular beds with continuous endothelium and post-capillary venules in lymphoid tissues and some inflamed non-lymphoid tissues [13]. However, the dynamic behavior of lymphocytes in peripheral non-lymphoid tissue such as the liver is largely unknown.

The accumulation of lymphocytes in liver sinusoids (devoid of E/P-selectins, PECAM, CD34, and VE-cadherin, and low in VCAM-1) appears to result from mechanisms distinct from those involved in multistep extravasation of intravascular lymphocytes [14]. Accumulation of CD1d-reactive T cells in the liver has been shown to require LFA-1 expression on liver cells other than NKT cells [15], which implicates hematopoietic cells such as Kupffer cells in capture of NKT cells since endothelial cells do not express LFA-1. However, all activated lymphocytes upregulate ICAM-1, so other mechanisms are needed to account for the large relative enrichment of NKT cells in liver compared to NKT cells in other sites and compared to other types of lymphocytes in the liver.

CXCR6/Bonzo/STRL33 is a chemokine receptor that can serve in conjunction with CD4 as a co-receptor for infection with some human and most simian immunodeficiency viruses (HIV-1, HIV-2, and SIV) [16] and, similarly to CCR5 and CXCR3, has an expression pattern restricted to memory/effector T cells such as NKT cells [17,18,19,20,21]. CXCR6 has one known ligand, CXCL16, a transmembrane chemokine expressed in spleen, lung, and liver, and on macrophages, dendritic cells, and some spleen sinus-lining cells [19,21,22].

In this investigation, we studied liver CD1d-specific NKT cells using mice in which the *cxcr6* coding region was replaced with a green fluorescent protein (GFP) cDNA. We demonstrate that CXCR6^+^ CD1d-reactive T cells crawl within liver sinusoids at speeds of 10–20 μm/min, and stop crawling upon TCR activation, revealing a novel form of intravascular immune surveillance. Interestingly, a complete deficiency in CXCR6 results in a selective and severe reduction of hepatic CD1d-reactive NKT cells, and is correlated with a decreased susceptibility to T-cell-dependent hepatitis. We observe that CXCR6 deficiency does not detectably alter the behavior of individual hepatic CD1d-specific NKT cells as they crawl along the CXCL16-expressing sinusoidal endothelial cells, but by regulating the number of hepatic NKT cells, at least in part by enhancing their survival, CXCR6 regulates liver-based immune responses.

## Results

### CXCR6^+^ T Cells Are Enriched in the Liver

**Figure 1:**
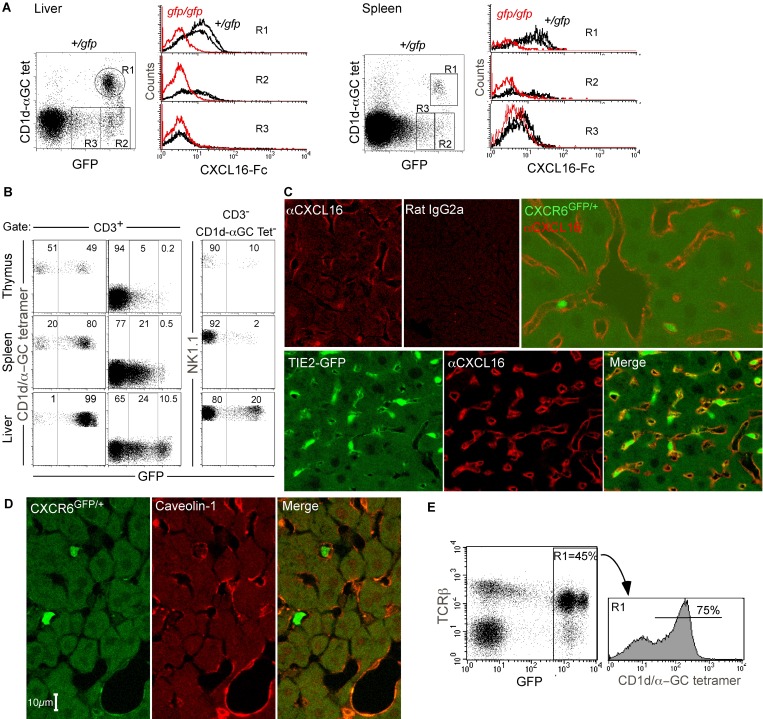
CXCR6^+^ Liver CD1d-Reactive T Cells Bind CXCL16 and Are Localized within CXCL16^+^ Liver Sinusoids (A) Binding of CXCL16 by splenocytes and liver leukocytes from *cxcr6^gfp/+^* and *cxcr6^gfp/gfp^* mice. Splenocytes and liver leukocytes from *cxcr6^gfp/+^* (+/−) and *cxcr6^gfp/gfp^* (−/−) mice were stained with PE-conjugated CD1d tetramer loaded with α-GalCer (αGC), PerCp-conjugated anti-CD3 antibodies and CXCL16-Fc fusion protein, and Cy5-conjugated goat anti-human Fc. The two black curves represent duplicate samples from two different *cxcr6^gfp/+^* mice. (B) Thymocytes, splenocytes, and liver leukocytes from *cxcr6^gfp/+^* and *cxcr6^gfp/gfp^* mice were stained with PE-conjugated CD1d tetramer loaded with α-GalCer and biotinylated anti-NK1.1 followed by PerCp-conjugated streptavidin and APC-conjugated anti-CD3 antibodies. Left panels are gated on CD3^+^ T cells and represent GFP fluorescence intensity on CD1d/α-GalCer-reactive T cells and on conventional (tetramer-negative) T cells. The right panels are gated on CD3^−^ cells and represent GFP fluorescence intensity on NK1.1^+^CD3^−^ NK cells. Numbers are percent obtained from a representative experiment among six performed. (C) Liver sinusoidal endothelial cells express CXCL16. Sections (10 μm thick) of PFA-fixed liver from wild-type mice (top panel, left), *cxcr6^gfp/+^* mice (top panel, right), and Tie2-GFP mice (bottom panel) were stained with rat monoclonal antibody against mouse CXCL16 (Clone 10H7, IgG2a), or with an isotype control, followed by goat anti-rat antibody (F[ab′]2) conjugated to Cy3. (D) GFP^hi^ CD1d-restricted T cells within liver sinusoids. Sections (7 μm thick) of PFA-fixed liver from *cxcr6^gfp/+^* mice were stained with rabbit polyclonal serum against caveolin-1 followed by goat anti-rabbit antibody conjugated to Cy3. (E) Flow cytometry analysis of cells harvested by perfusion of *cxcr6^gfp/+^* liver with cold PBS–ethylenediamine tetra-acetic acid.

GFP expression in *cxcr6^gfp/+^* mice is restricted to subsets of activated/memory T cells [20], including NKT cells [17,23]. CD1d-reactive NKT cells accounted for 75%–80% of GFP^hi^ cells in the liver (Figure 1A), and approximately half of GFP^hi^ cells in the spleen and other organs (Figure 1A; data not shown).

To confirm the correlation of GFP expression with that of CXCR6 at the plasma membrane on a per-cell basis, *cxcr6^gfp/+^* lymphocytes were analyzed for binding of CXCL16, the only known ligand of CXCR6, using a CXCL16-Fc fusion protein [19]. We detected CXCL16 binding in GFP^hi^ cells in *cxcr6^gfp/+^*, but not *cxcr6^gfp/gfp^* mice, in both spleen and liver, confirming that GFP^hi^ cells in heterozygous mice express the receptor and that its expression is bi-allelic (Figure 1A, R1- and R2-gated cells). We also observed that while 50% of thymic and 80% of splenic CD1d-tetramer-positive NKT cells expressed GFP, 99% of such cells expressed GFP/CXCR6 in liver (Figure 1B). Conventional T cells expressing CXCR6 were also found to be more frequent in liver than in other organs, and a subset of CD3^−^ NK cells expressing CXCR6 represented 20% of NK cells in the liver, but was not observed in other organs, including spleen, thymus, lung, and intestine (Figure 1B; data not shown).

### CXCL16, the CXCR6 Ligand, Is Expressed in Liver Sinusoids

We next examined the expression of the CXCR6 ligand, the transmembrane chemokine CXCL16, by immunofluorescence confocal microscopy of fixed tissue sections. CXCL16 has been shown by both RNA and protein analysis to be expressed in the spleen, lung, and liver [19,21,22]. Studies in mice have suggested that CXCL16 is expressed on dendritic cells and macrophages [19,22]. Staining of liver sections with a monoclonal anti-CXCL16 antibody showed a pattern consistent with CXCL16 expression on liver sinusoids and the contiguous centro-lobular venules, but not in portal tracts (Figure 1C, upper panels; data not shown). Anti-CXCL16 staining of liver from Tie2-GFP transgenic mice, which express GFP in endothelial cells, showed membrane localization of the CXCL16 signal in tight association with the cytoplasmic GFP signal (Figure 1C, lower panels), confirming that the chemokine is expressed in sinusoidal endothelial cells. A similar pattern of expression has been observed in human liver (M. Briskin, unpublished data).

### CXCR6^+^ NKT Cells Patrol Liver Sinusoids

We initially examined the anatomical localization of CXCR6^+^ T cells in the liver by immunofluorescence confocal microscopy of fixed tissue sections. Surprisingly, most GFP^+^ cells in the liver were localized in hepatic sinusoids, separated from hepatocytes by endothelial cells that stained with antibodies against caveolin-1 and CXCL16 (Figure 1C and 1D). Relatively few GFP^+^ cells were found in the portal tracts. To confirm that the GFP^+^ cells were in fact CD1d-reactive NKT cells, livers were perfused with cold phosphate-buffered saline (PBS)/2 mM ethylenediamine tetra-acetic acid to release cells in the vascular lumen. The effluent contained a number of GFP^hi^ cells similar to those obtained from the entire liver (data not shown), among which 75% were CD1d-α-GalCer-specific T cells (Figure 1E). It is also notable that within liver sinusoids there is a distinct population that expresses a very high level of GFP and binds CD1d-α-GalCer tetramer with moderate to low efficiency despite having normal expression of TCRβ (Figure 1A). This sub-population is unresolvable in *cxcr6^gfp/gfp^* mice, possibly as a consequence of bi-allelic GFP expression. We have not characterized this population further, but believe that it is a subset of NKT cells with reduced reactivity for CD1d-α-GalCer, possibly the non-Vα14 NKT cells that have been described elsewhere [7].

**Figure 2:**
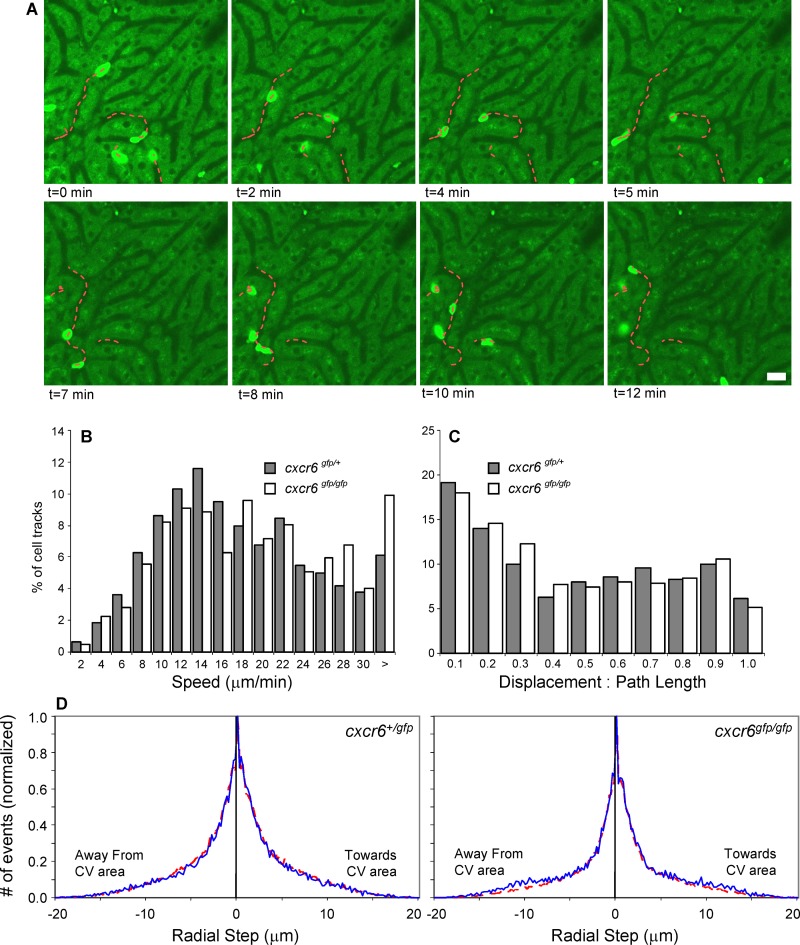
CXCR6^+^ Lymphocytes Patrol Liver Sinusoids (A) Select confocal microscopic images from intravital videos of liver of an anesthetized *cxcr6^gfp/+^* mouse (40× magnification). CD1d-reactive cells (bright green) can be seen migrating along hepatic sinusoids at an average speed of 16 μm/min. Scale bar is 25 μm. Cell tracks were traced and quantified using Volocity cell-imaging software. Note tracks of cells traveling in opposite directions in the same sinusoid (left side of image, at 0–5 versus 7–12 min). (B) Velocity quantification of GFP^+^ lymphocytes. Videos of liver in *cxcr6^gfp/+^* and *cxcr6^gfp/gfp^* mice (10× magnification) were analyzed for the velocity of GFP^+^ cells in 640 cell migration tracks for *cxcr6^gfp/+^* mice and 574 cell migration tracks for *cxcr6^gfp/gfp^* mice. Results demonstrate similar velocities of cells with the *cxcr6^gfp/+^* (filled bars, average velocity 16.5 ± 8.3 μm/min) and *cxcr6^gfp/gfp^* genotypes (unfilled bars, average velocity 18.4 ± 9.5 μm/min). (C) Analysis of directedness of cell migration. The same cell tracks as in (B) were analyzed for ratio of overall cell displacement to stepwise-summed path length. Results demonstrate similar degrees of directed crawling by cells from *cxcr6^gfp/+^* (filled bars, average 0.44 ± 0.31) and *cxcr6^gfp/gfp^* (unfilled bars, average 0.42 ± 0.30) mice. (D) Analysis of crawling relative to blood flow in peri-central vein areas of *cxcr6^gfp/+^* and *cxcr6^gfp/gfp^* mice. Histograms represent the frequency of cell movements made towards or away from nearby draining areas in likely close proximity to central veins (solid blue curve). The radial step of a cell is the cellular displacement along the axis defined by the cell's initial starting point to the central vein. The same step distances were assigned random orientations and the resultant data were plotted (red dashed curves).

To further understand the behavior of these sinusoidal NKT cells, we used fluorescence confocal imaging to observe the livers of live mice in real time (intravital microscopy). These experiments revealed that more than 90% of GFP^+^ cells in *cxcr6^gfp/+^* mice were stably attached to the sinusoidal wall. Time-lapse imaging revealed that these cells were actively crawling within the sinusoids (Figure 2A; Videos S1 and S2) with a mean speed of 16.5 ± 8.3 μm/min (Figure 2B; Video S3), more than 100-fold slower than typical rolling in post-capillary venules. Various examples of crawling patterns of hepatic NKT cells are shown in Video S4. Further analysis showed that crawling NKT cells choose their direction of motion largely randomly, and independently of the location of central veins or the direction of blood flow. This analysis required imaging at relatively high speeds (approximately 1 frame/s) after injecting fluorescent dextran intravenously, allowing us to clearly identify inlets and outlets corresponding to regions proximal to portal triads and central veins (Video S5). Moreover, cells were observed to pass each other in opposite directions in the same sinusoid and to reverse direction within a single sinusoid (see Videos S1 and S4). The mean ratio of cellular displacement over actual path length was 0.44 + 0.31 (Figure 2C).

The wide range of values reflects the branching nature of liver sinusoids and the chaotic nature of NKT cell migration, with some cells persisting in one direction for tens of microns, resulting in high values, and others frequently changing direction, resulting in low values. We determined whether GFP^+^ cells moved towards or away from central veins by plotting the radial step (the cellular displacement along the axis from the cell's initial point to the central vein) for each cell at each consecutive time point. In *cxcr6^gfp/+^* mice, steps towards and away from the central vein were equally frequent (Figure 2D, blue solid line), and the average radial step was nearly zero (0.02 μm away from the central vein). We also plotted a distribution attained when the angles for the same steps were randomized, and this curve (Figure 2D, red dashed curve) displayed a nearly perfect overlay with the experimental data (blue solid curve). A similar analysis for portal triads showed the same result (not shown). Thus, as a population, NKT cells choose their direction of motion largely randomly, and independently of the location of central veins or the direction of blood flow. We additionally conclude that there is no evidence for a haptotactic gradient of CXCL16 or any other ligand directing cells either towards or away from the central veins.

### NKT Cells Stop Patrolling upon TCR Activation

**Figure 3:**
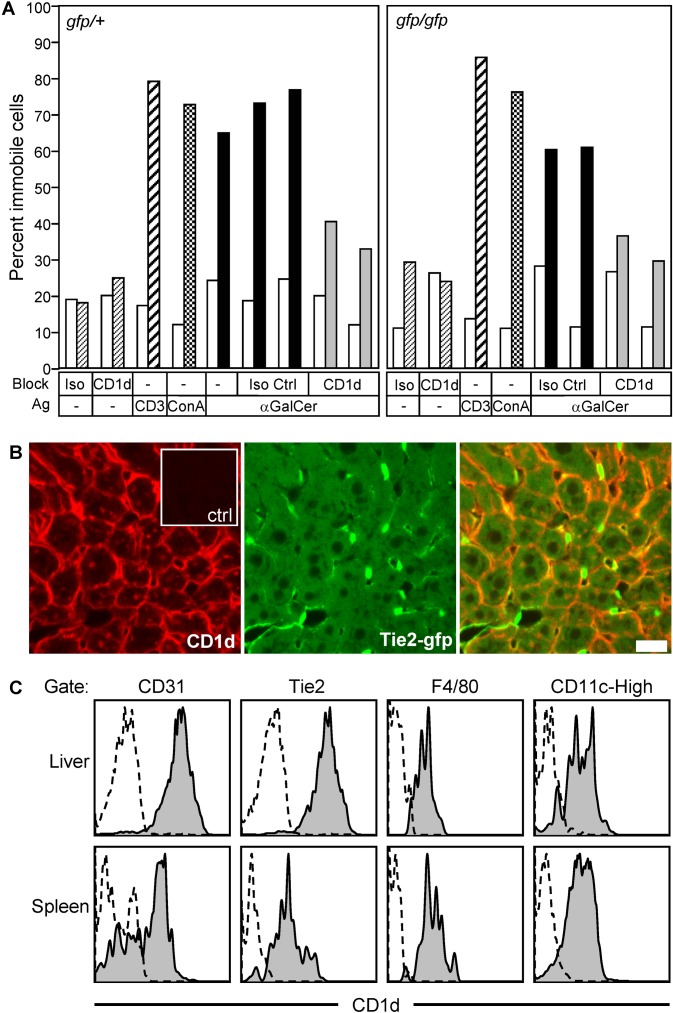
CXCR6^+^ Lymphocytes Stop Patrolling upon TCR Activation (A) Activation of NKT cells delivers stop signal. *cxcr6^gfp/+^* and *cxcr6^gfp/gfp^* mice were imaged before and after intravenous injection of antibody, lectin, or glycolipid antigen. Percentage of immobile cells was determined in 6-min videos either before any injection or 40 min after antigen delivery. (B) Histological examination of hepatic CD1d expression. Liver tissue from a Tie2-GFP transgenic was stained with anti-CD1d antibody (left panel, red) or an isotype control (inset). Green fluorescence indicates sinusoid-lining endothelial cells (expressing GFP) as well as the highly autofluorescent hepatocytes. (C) Flow-cytometric examination of hepatic CD1d expression. Cells isolated from liver tissue were stained with antibodies to endothelial cell markers (CD31 and Tie2), a Kupffer cell marker (F4/80), or a dendritic cell marker (CD11c) while co-stained with anti-murine CD1d (filled-in) or an isotype-matched control (dashed line).

By analogy to conventional T cells, which stop migrating in response to antigen in vitro [24], we hypothesized that TCR activation would modify the patrolling behavior of hepatic NKT cells. Indeed, following injection of anti-CD3ɛ antibody or ConA, 70%–90% of patrolling GFP^+^ cells arrested their movement within minutes (Figure 3A; Video S6). Injection of a control anti-CD1d antibody (clone 1B1) or of several T-cell-binding antibodies, including some matched to isotype of the anti-CD3 antibody, had no effect on patrolling (Figure 3A; data not shown). Intravenous delivery of the surrogate NKT cell antigen α-GalCer also triggered the arrest of most patrolling GFP^+^ cells, and was blocked by pre-administration of anti-CD1d antibody (Figure 3A; Video S7). These results further confirm that most patrolling GFP^+^ cells are CD1d-reactive T cells and indicate that these patrolling liver NKT cells undergo rapid arrest upon activation via TCRs.

### CD1d Is Expressed on Hepatic Sinusoid-Lining Cells

We sought to determine which hepatic cell types might be responsible for presenting α-GalCer to the intravascular NKT cells. Fluorescence microscopy of fixed tissue sections showed bright staining for CD1d in a pattern that was difficult to interpret (Figure 3B). The pattern suggests high expression on hepatocytes, including an intracellular peri-nuclear compartment, but whether other cell types also express CD1d was difficult to assess by this method. Using flow cytometry, we observed that sinusoid-lining endothelial cells (CD31^+^ cells and Tie2^+^ cells) express high levels of CD1d on their surface, and Kupffer cells (F4/80^+^) and dendritic cells (CD11c-high cells) express lower, but clearly detectable, levels of surface CD1d (Figure 3C). Thus, intravascular hepatic NKT cells have easy access to CD1d-expressing cells at all times.

### CD1d-Reactive T Cells Are Selectively Reduced in the Liver of CXCR6-Deficient Mice

To investigate a potential role for CXCR6 in patrolling, we also compared heterozygous *cxcr6^gfp/+^* mice to *cxcr6^gfp/gfp^* (i.e., CXCR6-deficient) littermates. We reasoned that because CXCL16 is a *trans*-membrane chemokine, CXCR6 could be involved in the intravascular movement of the CD1d-reactive T cells by affecting their adhesion or polarization during their migration within sinusoids. However, sinusoidal patrolling by GFP^+^ cells was similar in *cxcr6^gfp/+^* and *cxcr6^gfp/gfp^* mice; the speed (16.5 ± 8.3 μm/min versus 18.4 ± 9.5 μm/min; see Figure 2B and Videos S2 and S3), directedness (see Figure 2C), direction (see Figure 2D), and stopping (see Figure 3A) were unaltered. Our data thus do not support a critical role of CXCR6 in general crawling behavior of liver NKT cells.

**Figure 4:**
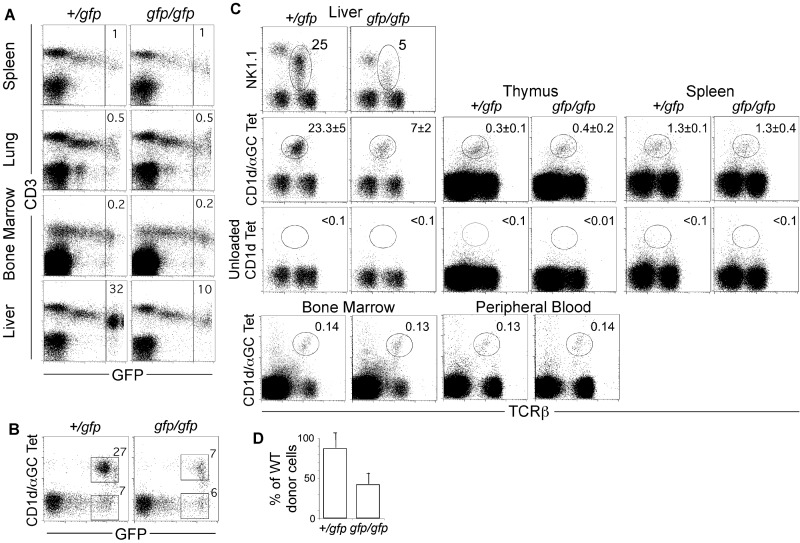
CD1d-Reactive NKT Cells Express CXCR6, Are Enriched in Liver, and Are Selectively Reduced in CXCR6-Deficient Mice Splenocytes, thymocytes, and lung, bone marrow, blood, and liver leukocytes from *cxcr6^gfp/+^* (+/−) and *cxcr6^gfp/gfp^* (−/−) mice were stained with one of the following combinations: K1.1 PE-conjugated antibodies and APC-conjugated anti-TCRβ; PE-conjugated CD1d tetramer loaded with α-GalCer (αGC) and APC-conjugated anti-TCRβ; or control unloaded tetramer and APC-conjugated anti-TCRβ. (A) Expression of CD3 and GFP by cells from different organs. (B) Reduction in CD1d -α-GalCer-tetramer-positive liver leukocytes in *cxcr6^gfp/gfp^* mice. (C) Selective reduction of CD1d-reactive T cells in the liver of CXCR6-deficient mice. Results are mean ± standard deviation from four experiments. (D) Reduced ability of CXCR6-deficient NKT cells to accumulate in the liver of recipient mice. Thymocytes from *cxcr6^+/+^* mice were co-transferred with thymocytes from either *cxcr6^gfp/+^* or *cxcr6^gfp/gfp^* littermates into TCRα-deficient mice. The phenotype of hepatic leukocytes 2 d after transfer is shown. Similar results were obtained at 3 days. The results shown are representative of three experiments.

In comparing the heterozygous to the homozygous mutant mice, however, we noticed a significant reduction in the number of GFP^+^ NKT cells in livers from the CXCR6-deficient animals. Flow cytometry revealed 3- to 5-fold fewer GFP^hi^ CD3^+^ TCRβ^+^ CD1d-reactive NKT cells in livers from homozygous null versus heterozygous mice (Figure 4A–4C). Notably, GFP^+^ CD1d-reactive tetramer-binding T cells in the thymus, peripheral blood, spleen, bone marrow, and lung were found in similar numbers in *cxcr6^gfp/+^* and *cxcr6^gfp/gfp^* littermates (Figure 4C; data not shown). GFP^+^ and GFP^−^ CD44^hi^ conventional effector/memory T cells and NK1.1^+^ CD3^−^ NK cells were also found in similar numbers in the liver, thymus, peripheral blood, spleen bone, marrow, lung, and small intestine of *cxcr6^gfp/+^* and *cxcr6^gfp/gfp^* littermates (data not shown). No significant difference in number of hepatic CD1d-reactive NKT cells was observed between wild-type and *cxcr6^gfp/+^* mice (data not shown).

To prove that this defect was T cell intrinsic, 1.5 × 10^7^ thymocytes from 4-wk-old *cxcr6^+/+^* mice were mixed with equal numbers of thymocytes from *cxcr6^gfp/+^* or *cxcr6^gfp/gfp^* littermates and co-transferred intravenously into *tcrα^−/−^* recipients devoid of TCR*α*β lymphocytes. After 2–3 d, donor-derived *cxcr6^gfp/gfp^* NKT cells were underrepresented in the recipient liver in comparison to cells from heterozygous or wild-type control mice (Figure 4D). Together, these findings demonstrate that CXCR6 has a role in mediating the accumulation in liver of CD1d-reactive NKT cells.

### CXCR6 Expression Prolongs Survival of CD1d-Reactive T Cells

**Figure 5:**
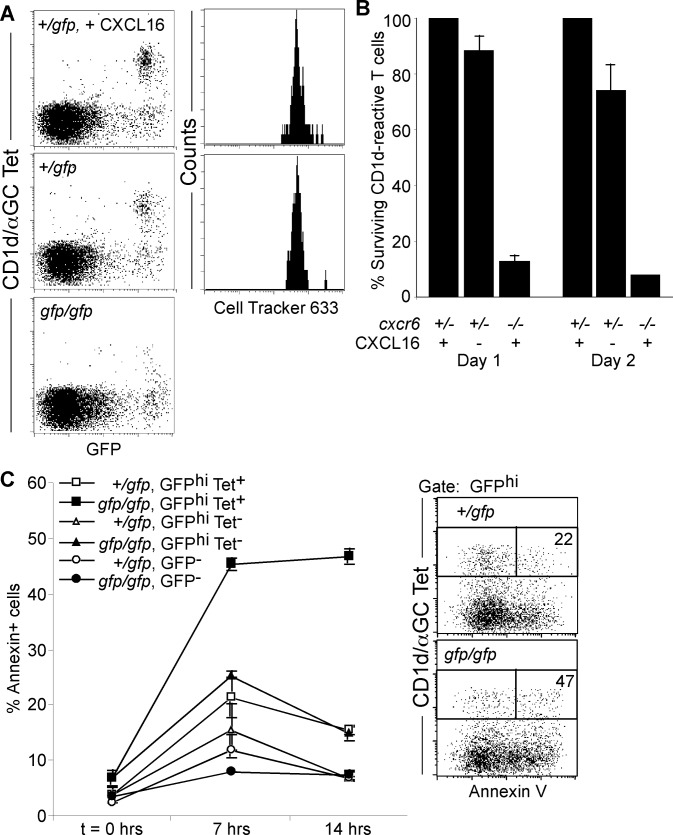
Requirement for CXCR6 in Survival of CD1d-Reactive T Cells (A) Flow cytometry analysis of liver leukocytes from GFP knock-in mice after overnight culture. Dot plots represent GFP signal versus CD1d-α-GalCer-tetramer staining. Histogram plots represent Cell Tracker 633 labeling gated on tetramer-positive cells, indicating no cell division during the course of the culture. (B) Survival of liver NKT cells from *cxcr6^gfp/+^* and *cxcr6^gfp/gfp^* mice. Duplicate samples of 5 × 10^4^ CD1d-reactive T cells/well were incubated for each condition and time point. Histograms represent the percentage of viable, CD1d-α-GalCer-tetramer-positive GFP^hi^ cells, as determined by flow cytometry at the indicated time points. The proportion of CXCL16-cultured cells from control *cxcr6^gfp/+^* mice was set at 100%. Values are mean ± standard deviation from three independent experiments. (C) Flow cytometry analysis of liver leukocyte apoptosis in culture. The percentage of cells binding Annexin V was determined by flow cytometry at the indicated time points. Results are representative of two independent experiments. A representative analysis at 10 h is shown on the right, with the percentage of CD1-αGC-tetramer-positive and Annexin V–positive cells indicated.

Because CXCR6 deficiency appeared to not alter the steady-state behavior of crawling liver NKT cells, we hypothesized that their reduced number may be caused by their impaired survival or decreased proliferation. Staining of liver sections with antibody specific for activated caspase-3 showed only 1%–2% apoptotic cells among GFP^hi^ cells in *cxcr6^gfp/+^* and *cxcr6^gfp/gfp^* mice, and Annexin V staining of CD1d-reactive GFP^hi^ cells by flow cytometry gave similar results (data not shown). This may reflect a slow rate of apoptosis, rapid scavenging of apoptotic cells by Kupffer cells [25,26], or detachment of apoptotic NKT cells from the liver sinusoids in vivo. We therefore examined apoptosis ex vivo, by culturing Percoll gradient-purified liver mononuclear cells from *cxcr6^gfp/+^* and *cxcr6^gfp/gfp^* mice for various time periods in 96-well plates. The number of GFP^hi^-tetramer-positive T cells from *cxcr6^gfp/gfp^* mice, recovered after 18 h in culture, was dramatically reduced in comparison with that from *cxcr6^gfp/+^* mice (Figure 5A and 5B). Consistent with this observation, Annexin V staining of CD1d-reactive GFP^hi^ cells isolated from liver indicated that *cxcr6^gfp/gfp^* cells underwent apoptosis more rapidly than *cxcr6^gfp/+^* cells (Figure 5C). CD1d-reactive T cells did not proliferate ex vivo, as evaluated by staining with Cell Tracker 633 (Figure 5A), suggesting that impaired survival rather than reduced proliferation contributes to the reduction in CD1d-reactive NKT cells recovered from the CXCR6-deficient mice. Addition of exogenous CXCL16 to the ex vivo cultures resulted in a significant enhancement of survival of *cxcr6^gfp/+^* cells, but it is worth noting that even without exogenous CXCL16, *cxcr6^gfp/+^* cells survived longer ex vivo than *cxcr6^gfp/gfp^* cells (Figure 5B), suggesting that the pro-survival consequences of CXCR6 signaling can persist for long periods of time (days). However, it is possible that contaminating endothelial cells or soluble CXCL16 in the fetal bovine serum provided additional CXCR6 stimulation during the ex vivo incubation.

### Decreased ConA-Induced Hepatitis and Reduced Sinusoid Patrolling by NKT Cells in CXCR6-Deficient Mice

**Figure 6:**
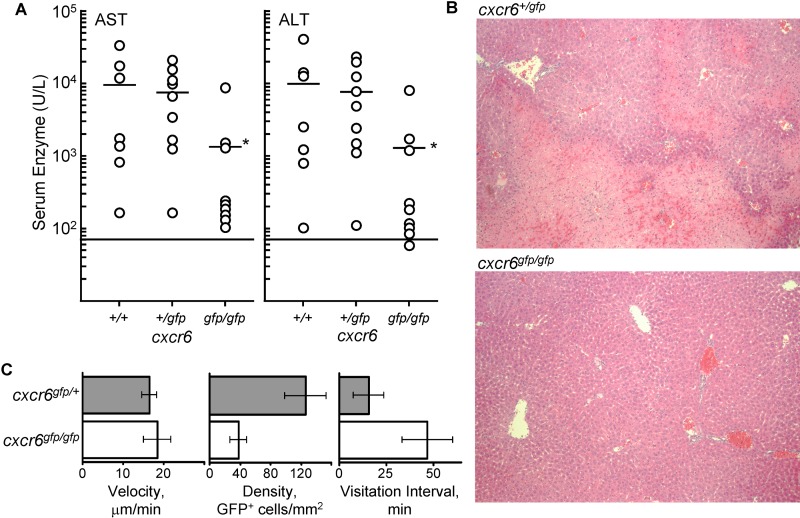
Decreased Patrolling Efficiency and Decreased ConA Hepatitis Severity in CXCR6-Deficient Mice (A) Serum transaminase levels in *cxcr6^gfp/gfp^*, *cxcr6^gfp/+^*, and *cxcr6^+/+^* littermates 12 h after a challenge with 20 mg/kg ConA IP. Results are pooled from two independent experiments. The horizontal line represents the upper limit for normal serum transaminase levels, as measured in unchallenged mice. Asterisk indicates a two-tailed student's *t*-test with a *p* < 0.05 in comparison with littermates. (B) Hematoxylin and eosin staining of paraffin-embedded liver sections from *cxcr6^gfp/+^* and *cxcr6^gfp/gfp^* mice sacrificed 12 h after a challenge with 20 mg/kg ConA IP. (C) Reduced sinusoid patrolling by NKT cells in *cxcr6^gfp/gfp^* mice. By measuring the average inter-nuclei distance of hepatocytes in high-magnification images, the average sinusoidal length of a hepatocyte was determined to be 28.6 μm (data not shown). Utilizing the lymphocyte velocity data (left panel) and assuming that a CD1d-reactive T cell can contact only one hepatocyte at a time, we calculated that each CD1d-reactive T cell can visit 0.58 hepatocytes/min in the *cxcr6^gfp/+^* mice, and 0.64 hepatocytes/min in the CXCR6-deficient mice. The density of GFP^+^ cells in *cxcr6^gfp/+^* and *cxcr6^gfp/gfp^* mice (middle panel) was calculated from the intravital videos used in Figure 3 and the flow cytometry data in Figure 2. Combining the similar crawling velocities with the different steady-state densities, we show decreased rate of patrolling by GFP^+^ lymphocytes, expressed as the average time between “visitation” of any single hepatocyte (right panel).

The reduction in the number of patrolling CD1d-reactive T cells in the CXCR6-deficient mice may be expected to decrease the efficacy of their effector functions. ConA-induced hepatitis, considered to be an experimental model for human autoimmune hepatitis [11], is strictly dependent on CD1d-reactive NKT cells [9,10]. We injected ConA into *cxcr6^gfp/gfp^*, *cxcr6^gfp/+^*, and *cxcr6^+/+^* littermates and measured serum aspartate aminotransferase (AST) and alanine aminotransferase (ALT) levels at several time points. Elevated serum levels of AST and ALT were first observed 6–8 h after injection of 20 mg/kg of ConA and peaked at 12 h after injection (data not shown). As shown in Figure 6A, serum AST and ALT levels were markedly reduced at 12 h in *cxcr6^gfp/gfp^* mice as compared with *cxcr6^gfp/+^* and *cxcr6^+/+^* littermates. Histological examination of livers from ConA-treated *cxcr6^gfp/+^* and *cxcr6^+/+^* mice showed severe bridging necrosis 12 h after injection in the area between the central veins and the portal tracts (Figure 6B). Numerous red blood cells were also observed in the sinusoidal area. In *cxcr6^gfp/gfp^* mice, the histological features of hepatitis were markedly less severe than in the *cxcr6^gfp/+^* littermates (Figure 6B). Thus, the reduced presence of CD1d-specific NKT cells in CXCR6-deficient mice was correlated with a decreased severity of acute hepatitis. Interestingly, the decrease in severity of hepatitis (an approximately 8-fold decrease in AST and ALT levels) appeared greater than the decrease in NKT density or visitation rate (3-fold). This is possibly due to cumulative effects of hepatic damage that tend to exacerbate each other, such as local damage leading to local ischemia, causing further damage.

The reduction in the number of CD1d-reactive T cells in the CXCR6-deficient mice might also be expected to decrease the efficiency of patrolling. Based on the results shown in Figure 2 and in the intravital videos (e.g., Videos S2 and S3), we found little difference in the rate of surveillance of sinusoids by individual CD1d-reactive T cells in *cxcr6^gfp/+^* and *cxcr6^gfp/gfp^* mice. The cells in *cxcr6^gfp/+^* and *cxcr6^gfp/gfp^* mice “visited” 0.58 and 0.64 hepatocytes/min, respectively (Figure 6C). However, because of the reduced density of CD1d-reactive T cells in *cxcr6^gfp/gfp^* mice, we estimate that each hepatocyte is visited only once every 47 min, as compared to every 15 min in *cxcr6^gfp/+^* mice. Therefore, the low number of CD1d-reactive T cells in the CXCR6-deficient mice clearly decreases the efficiency of patrolling.

## Discussion

This study illuminates several important aspects of the biology of CD1d-reactive NKT cells in vivo and provides a new paradigm for effector lymphocyte action in the liver. We have shown that CD1d-reactive NKT cells patrol liver sinusoids, the vascular space of the liver, rather than extravasate like effector cells in other tissues. The recruitment of NKT cells to the vascular endothelium in the liver thus results in intravascular patrolling rather than cellular extravasation. Patrolling appears to be a search for antigen, since it ceases following antigen receptor signaling. The chemokine receptor CXCR6 regulates the number of hepatic NKT cells, at least in part by transmitting survival signals.

### CD1d-Reactive NKT Cells Patrol Hepatic Sinusoids

By replacing the murine *cxcr6* gene with the coding region for GFP, we were able to visualize CXCR6-expressing cells throughout the body. GFP^hi^ cells were mainly located intravascularly in mouse liver sinusoids, and by intravital fluorescence microscopy they were found to be crawling within the sinusoids at high speeds. This patrolling behavior appears to be independent of the direction of blood flow. Activation through the TCRs delivered a rapid stop signal to these cells, indicating that the patrolling is a form of immune surveillance.

The only prior indication for patrolling in the liver was the observation of cells crawling within the sinusoids when viewed by dark-field oblique transillumination microscopy [27]. These cells were thought at the time to be Kupffer cells (liver macrophages). However, closer examination suggests that two populations were observed: a phagocytic non-motile population, probably Kupffer cells, and a non-phagocytic, rapidly migrating cell type that probably corresponds to the CD1d-reactive NKT cells examined here, though other effector populations such as NK cells and cytotoxic T lymphocytes may also be capable of patrolling the liver from within the sinusoids.

The finding of TCR^+^ cells that survey tissue for antigen while located intravascularly is unprecedented. Immune surveillance within lymph nodes and the spleen occurs in specialized compartments densely packed with lymphocytes, which are shielded from the high flow rates and shear forces of blood. In contrast, within the liver, hepatocytes, sinusoid-lining endothelial cells, hepatic stellate cells (Ito cells), and Kuppfer cells are all in direct contact with blood. Hence, intravascular patrolling by T cells is likely to be the most efficient means for detection of hepatic antigens.

We estimate that, at steady state, the area around each hepatocyte is visited by approximately four CD1d-reactive T cells/h. In stark contrast, dendritic cells in lymph nodes were recently estimated to be scanned by 5,000 T cells/h [28]. However, NKT cells have a highly restricted TCR repertoire, whereas naïve T cells are extraordinarily diverse. Thus, discovery of antigen may in fact be more rapid in the liver than in the lymph nodes, despite a 1,000-fold difference in the visitation rate of antigen-presenting cells.

NKT cells may be particularly well suited for surveillance of the liver because of the extensive lipid metabolism in this organ by hepatocytes, Kupffer cells, hepatic stellate cells, and the sinusoid-lining endothelial cells. CD1d is expressed on hepatocytes, Kupffer cells, hepatic dendritic cells, and sinusoid-lining endothelial cells (see Figure 3C), all of which have been demonstrated to have significant antigen-uptake capabilities [1]. It is unclear whether intravascular NKT cells could contact CD1d on hepatocytes, which are separated from the sinusoid by the sinusoid-lining endothelial cells. Direct contact might be achieved through the approximately 100-nm fenestrations typical of these endothelial cells. Alternatively, CD1d-reactive T cells may survey only the endothelium or Kupffer cells for inflammatory signals and/or for CD1d-bound endogenous ligands.

We have previously shown that CD4^+^ effector T cells stop migrating in response to antigen in vitro [24]. Here we have shown that NKT cells, essentially a population of effector T cells, display rapid and sustained stopping following TCR activation. In contrast, recent studies have shown that naïve T cells in lymph nodes continue to move at high speed for several hours after initial encounter with antigen-presenting dendritic cells, forming only short-lived conjugates, while only at later times after initial antigen encounter (8–20 h) did the T cells form long-term conjugates (>1 h) with dendritic cells [29,30].

The cytotoxic function of NKT cells has been implicated as necessary for ConA-induced hepatitis [9], although the observation that ConA triggers stopping of patrolling appears to argue against direct NKT-cell-mediated killing. NKT cell activation and subsequent cytokine production are known to recruit and activate large numbers of NK cells [31], which may instead be responsible for the direct killing. It is also possible that NKT cells resume patrolling after several hours of immotility, and in this way regain access to all the hepatocytes of the liver for direct killing. Further experiments will be required to resolve these questions.

### CXCR6 Controls the Accumulation of CD1d-Reactive NKT Cells in the Liver

Our study shows that CXCR6, which is expressed on CD1d-reactive NKT cells, controls the selective accumulation of these cells in the liver. Accordingly, CXCR6 also influences the ability of CD1d-reactive NKT cells to induce hepatitis caused by ConA. According to the multistep paradigm of lymphocyte extravasation, chemokines play a key role in activating adhesion molecules that transform rolling cells to firmly adhered ones. Because we observed that CXCL16, the chemokine ligand for CXCR6, is expressed on sinusoidal endothelial cells, we initially hypothesized that CXCR6 was critical for the recruitment of blood-borne NKT cells to the hepatic sinusoids, either by initial tethering interactions or by signaling to initiate crawling and patrolling. Utilizing high-frame-rate imaging, such recruitment events were occasionally observed (see Video S8). Many hours of observation showed no significant difference in the frequency of these recruitment events between *cxcr6^gfp/+^* and *cxcr6^gfp/gfp^* mice (data not shown), but the limited number of events and mouse-to-mouse variability preclude definitive conclusions regarding the role of CXCR6 in this process. In addition, we did not observe any significant deficit in hepatic recruitment of CXCR6-deficient cells after short-term transfer of T cell blasts from *cxcr6^gfp/+^* and *cxcr6^gfp/gfp^* mice and after in vivo expansion of NKT cells with α-GalCer treatment (data not shown). These results suggest the existence of redundant mechanisms for NKT cell recruitment to liver sinusoids. We have therefore been unable to support the hypothesis that a defect in the NKT cell recruitment process contributes to the phenotype observed at steady state in CXCR6-deficient animals.

Our results indicate that the CXCR6-deficient hepatic NKT cells patrol in the same manner as their CXCR6-expressing counterparts: they both crawl rapidly, lack directional bias, and rapidly stop in response to TCR stimulation. Thus, there is no defect in the patrolling behavior of CXCR6-deficient NKT cells that can explain their reduced numbers in the liver.

The reduced accumulation of CXCR6-deficient CD1d-reactive NKT cells in the liver is likely to result at least partially from increased cell death due to the lack of a CXCR6-mediated survival signal. Because CXCL16 is the only known ligand for CXCR6 and is expressed on liver sinusoids, it is reasonable to hypothesize that CXCR6/CXCL16 interactions result in enhanced survival of CD1d-reactive liver NKT cells. This would be a novel role for chemokines in the homeostasis of effector T cells in peripheral tissues. As CXCR6 is expressed not only on NKT cells, but also on activated and memory T cells, it is possible that this is a general mechanism by which chemokines mediate the survival of effector/memory T cells that patrol at sites of potential toxic damage and antigen entry, thus facilitating rapid and efficient memory T cell responses.

The nature of the survival signal provided by CXCR6 remains to be studied. Although unexpected, this behavior is not unprecedented, as CXCR4 and CX_3_CR1 have been reported to mediate survival in certain conditions [32,33,34]. Reports have suggested that Akt activation following ligand binding to the chemokine receptors CXCR4 and CX_3_CR1 results in enhanced survival of cells [32,33], and it has recently been shown that CXCR6 can similarly activate Akt [35]. It is also noteworthy that the defect in CXCR6 expression only affects hepatic NKT cells, and not NKT cells in other locations, suggesting that either the liver is an especially inhospitable environment, or that other survival signals are provided in other NKT-rich organs such as the spleen. The former seems a particularly attractive hypothesis since there is evidence for the liver being a pro-apoptotic environment for lymphocytes [1].

The results of this paper lead us to a model in which the number of intravascular patrolling NKT cells, which is regulated by CXCR6–CXCL16 interactions, influences the immune response in liver in two important ways: (1) it determines the frequency of new antigen detection by affecting the visitation rate of parenchymal liver cells, and (2) it governs the total cytokine “power” (pooled secretion capacity) of the hepatic NKT cell population. In the case of the ConA hepatitis model, the first factor is non-applicable because the “antigen” is in excess. However, in infectious pathogen models in which rare hepatocytes may be infected, there are likely to be agonist glycolipids that react with only a small subset of NKT cells and thus may escape detection for hours, or even days, if NKT cell density is too low. Potential examples include the recent finding that small subsets of NKT cells recognize the mycobacterial cell-wall antigen PIM_4_ [36] and lipophosphoglycan of leishmania [37].

In conclusion, we have described a model that may explain how the immune system monitors the status of the liver. Future experiments will be required to identify the molecules involved in both NKT cell crawling and stopping processes and the cells that are surveyed by NKT cells, and to determine the relevance of this system for the pathogenesis of hepatitis and for various immune responses in the liver.

## Materials and Methods

### 

#### Animals

*cxcr6^gfp/+^* knock-in mice were generated as described [20] and backcrossed three to eight times onto C57BL/6. Tie2-GFP mice on the FVB/NJ background and *tcrα^−/−^* mice on the C57BL/6 background were purchased from Jackson Laboratory (Bar Harbor, Maine, United States) and Taconic Farms (Germantown, New York, United States), respectively. All mice were maintained in our specific-pathogen-free animal facility according to institutional guidelines, and experiments were done with mice from 4 to 12 wk of age.

#### Reagents

CD1d tetramer loaded with α-GalCer was prepared as described previously [6]. The α-GalCer compound DB01–1 was kindly provided by Steven Porcelli. The CXCL16-Fc fusion protein was a kind gift of J. Cyster. Recombinant murine CXCL16, SDF-1, and fractalkine were purchased from R & D Systems (Minneapolis, Minnesota, United States). ConA was purchased from Sigma-Aldrich (St. Louis, Missouri, United States). The following monoclonal antibodies were purchased from PharMingen (San Diego, California, United States): H57–597-PE and -APC (anti-TCRβ); PK136-PE, -biotin, and -PE (anti-NK1.1); IM7-biotin (anti-CD44); and 2.4G2 (anti-FcγRII/III). Rabbit polyclonal serum against caveolin-1 was purchased from Transduction Laboratories (Lexington, Kentucky, United States). Goat F(ab′)2 anti-human IgG Fc conjugated to Cy5, goat F(ab′)2 anti-rat IgG Fc conjugated to Cy3, and goat anti-rabbit Ig conjugated to Cy3 were purchased from Jackson ImmunoResearch (West Grove, Pennsylvania, United States). Anti-CD1d antibody (clone 1B1) was purchased from PharMingen and conjugated to Cy5 succinimide ester (Amersham Biosciences, Little Chalfont, United Kingdom) at a final ratio of 1.7 fluorophores/antibody. Cell Tracker 633 (Bodipy 630/650 MeBr) was purchased from Molecular Probes (Eugene, Oregon, United States).

#### Production of anti-murine CXCL16 antibodies

Female Wistar-Kyoto rats, 6–8 wk old, were immunized intraperitoneally with 100 μg of murine CXCL16. Immunizations were performed with 100 μg of protein emulsified in incomplete Freund's adjuvant at 2-wk intervals. After a minimum of three immunizations, the rats were boosted with 100 μg of soluble CXCL16 protein in PBS. Three days post-boost, the spleens were harvested and fused with SP2/0 myeloma cells. The fusion was screened by ELISA using plates coated with CXCL16 protein. Specificity of the hybridomas was determined by ELISA with a panel of murine chemokines including CXCL11, CCL22, CCL5, CCL27, CCL28, CCL17, CXCL10, CCL25, and CXCL9. Hybridomas producing CXCL16-specific antibodies were then subjected to three rounds of subcloning by limiting dilution. Biological activity was confirmed by FACS and inhibition of chemotaxis.

#### Isolation and staining of lymphocytes from mouse tissues

Spleen tissue was minced and mashed through a 70-μm strainer in PBS with 0.5% BSA. The resulting suspension was pelleted by centrifugation, re-suspended in PBS with 0.5% BSA, layered on Ficoll-paque (Pharmacia LKB Technology, Uppsala, Sweden), and centrifuged at 400 *g* for 20 min. Blood was collected in heparinized tubes by cardiac puncture of anesthetized animals. Bone marrow was obtained by flushing femurs with PBS containing 0.5% BSA using a 26 G needle. Cells in the resulting pellets were treated with tris-ammonium chloride to remove red blood cells and then washed extensively before use. Lung or liver tissue was minced and mashed through a 70-μm strainer in PBS containing 0.5% BSA. The resulting suspension was pelleted by centrifugation, re-suspended in 40% Percoll (Pharmacia LKB Technology), layered on 80% Percoll, and centrifuged at 600 *g.* Cells at the gradient interface were harvested and washed extensively before use. Alternatively*,* liver mononuclear cells were recovered by perfusion of liver of anesthetized mice as follows: the diaphragm and the sus-hepatic vein were cut and the liver reclined in a Petri dish; perfusion with cold PBS and 2 mM EDTA was through the aorta, and the effluent was collected through the severed sus-hepatic vein (approximately 5 ml).

#### Staining of CD1d on parenchymal hepatic cells

Fluorescence microscopy of fixed liver sections was performed as described above for CXCL16 analysis. For flow cytometry analysis, liver tissue was mashed with the back of a 5-ml syringe plunger, digested in PBS containing Mg and Ca by 0.2 mg/ml Collagenase D (Roche, Basel, Switzerland) and 0.02 mg/ml DNase I (Roche) for 30 min at 37 °C while agitating, filtered through a 70-μm strainer, pelleted in 40% Percoll (Pharmacia LKB Technology), cleared of red blood cells by tris-ammonium chloride solution, and washed with cold PBS. This suspension of hepatic parenchymal cells was blocked with goat and bovine serum (2% each) and anti-CD16 antibody (PharMingen) for 20 min on ice and then stained with CD31-PE or Tie2-PE (eBioscience, San Diego, California, United States), F4/80-PE (Serotec, Oxford, United Kingdom), or CD11c-PE (PharMingen) along with anti-murine CD1d-Cy5 or rat IgG2b-Cy5. After 30 min on ice, cells were washed twice and analyzed by flow cytometry.

#### Thymocyte transfer experiment

Thymocytes were isolated from 4-wk-old *cxcr6^+/+^*, *cxcr6^gfp/+^*, and *cxcr6^gfp/gfp^* littermates. *tcrα^−/−^* recipient mice (devoid of TCRαβ lymphocytes) were grafted IV with 30 × 10^6^ thymocytes from a 50:50 mix of cells (*cxcr6^+/+^*:*cxcr6^gfp/+^* and *cxcr6^+/+^*:*cxcr6^gfp/gfp^*). After 2 and 3 d, recipient mice were sacrificed, and liver cell suspensions were prepared and stained with antibodies against TCRβ-APC, and NK1.1-PE or CD1d-*α*-GalCer-tetramer-PE. After exclusion of dead cells with PI, cells present in the TCRβ^+^ gate were analyzed for staining with NK1.1 or CD1d tetramer and GFP.

#### ConA hepatitis

During preliminary time-course and dose-escalation experiments using wild-type and TCRα KO 6-wk-old C57BL6 mice, a dose of 20 mg/kg ConA IP was determined to induce acute liver disease with a peak in serum transaminases AST and ALT at 12 h. Groups of 6-wk-old *cxcr6^+/+^*, *cxcr6^gfp/+^*, and *cxcr6^gfp/gfp^* mice were therefore treated with 20 mg/kg ConA diluted in PBS, blood was sampled by tail bleeding after 12 h, serum was aliquoted and stored at −20 °C, and transaminases were measured using a Vitros 950 (Ortho-Clinical Diagnosis, Mississauga, Ontario, Canada).

#### In vitro survival/proliferation assay

Liver leukocytes were isolated from 8- to 12-week-old *cxcr6^gfp/+^* and *cxcr6^gfp/gfp^* littermates. Equal numbers of CD1d-α-GalCer-tetramer-PE-positive T cells, as measured by flow cytometry, were cultured in RPMI 1640 medium with 10% FCS, 1% penicillin/streptomycin, and beta-mercapto-ethanol on 96-well plates coated sequentially with 0.5 μg/well anti-CD3 antibody (2C11, PharMingen) and/or 0.5 μg/well recombinant CXCL16 diluted in PBS or with PBS alone. The number of GFP^hi^ CD1d-α-GalCer-tetramer-PE-positive T cells, surface-stained with Annexin V (PharMingen), were determined in duplicate, at the indicated time points, by flow cytometry. The number of GFP^hi^ CD1d-α-GalCer-tetramer-PE-positive T cells in the sample from *gfp/+* mice cultivated in wells coated with CXCL16 was considered to be 100%.

#### Confocal microscopy

Livers were dissected and removed, washed in PBS, sliced, and fixed for 45min at 4 °C in 4% paraformaldehyde. Liver slices were washed with PBS and incubated overnight at 4 °C in 30% sucrose, and then washed again in PBS, placed in OCT medium, and frozen. Sections with a thickness of 7 μm were stained with rabbit polyclonal anti-caveolin-1 serum followed by goat anti-rabbit antibody conjugated to Cy3, with rat monoclonal IgG2b anti-CXCL16 followed by goat anti-rat antibody conjugated to Cy3, and with control serum and isotype control antibodies followed by the corresponding second-step reagents. Slides were mounted with Fluoprep (Biomerieux SA, Marcy l'Etoile, France) and analyzed with a confocal laser system (LSM 510, Zeiss, Jena, Germany).

#### Intravital confocal microscopy

Surgical preparation for liver imaging was based on methods described previously [38]. Mice were anesthetized using a cocktail of ketamine (50 mg/kg), xylazine (10 mg/kg), and acepromazine (1.7 mg/kg) injected intraperitoneally. Beginning 45 min later, anesthesia was maintained by half-dose boosts subcutaneously every 30 min. Hair from the left subcostal region was trimmed, and the liver was exposed through a 1.5-cm horizontal incision. The hepatoform ligament was cut and the tip of the left lobe of the liver gently extruded. The mouse was inverted onto a pre-prepared plastic or aluminum tray in which a coverslip was mounted near the center and narrow strips of paper (1.5 mm × 1.5 cm) were glued. These strips of paper provided friction that helped to immobilize the tissue being imaged. Images were acquired using an inverted epifluorescence Zeiss LSM 510 confocal laser system equipped with a 10×/0.3 Plan Neofluar objective, or Fluar 40×/1.3 objective. A warming fan blowing into an enclosure around the whole microscope was used to keep the area warm, and the microscope objective was thermostatically controlled to maintain 37 °C. Videos were acquired by consecutive frames using appropriate combinations of 488-nm, 546-nm, and 633-nm laser lines and GFP, Cy3, and Cy5 filter sets. Imaging speed varied between videos with a range from 1 to 30 s per time point. During all of our intravital imaging experiments, we were keenly aware of the possibility of local photo-toxicity causing time-dependent artifacts in our data. However, we were unable to detect, either by eye or by quantitative analysis, any indication of such a phenomenon.

#### In vivo activation and imaging of NKT cells

Animals were imaged as described above. Antibodies and antigens were injected in 50 μl of PBS solution containing 100 μg of 70-kDa dextran conjugated to tetramethylrhodamine (Molecular Probes) to confirm intravenous delivery and healthy blood flow in the region being imaged. We injected 5 μg of anti-CD3ɛ (2C11 clone, PharMingen), 250 μg of Alexa-633-conjugated ConA (Molecular Probes), or 0.05–5 μg of α-GalCer (DB01–1 compound, kind gift of S. Porcelli). Initial experiments utilized 5 μg of α-GalCer, but as little as 50 ng was sufficient to trigger identical amounts of NKT stopping. Doses of either 200 or 50 ng of α-GalCer were used in the GK1.5/CD1d blocking experiments shown here. The percentages of immobile cells before and 40 min after injection of antigen were assessed by manual observation: 6 min of video was analyzed, and cells that moved less than one cell-body-length (10 μm) were considered immobile.

#### Analysis of intravital imaging videos

Quantitative analysis was performed only on videos in which there was no detectable whole-liver movement. Cells were tracked using Volocity software version 2.0 (Improvision, Lexington, Massachusetts, United States). Because the software lost some cells due to cells moving out of focus or coming close to one another, the resulting data were an array of cell paths ranging from single frames to the full length of the video (typically 10–15 min). No effort was made to reassemble these partial tracks, but there appeared to be no significant difference in the distribution of track lengths between *cxcr6^gfp/+^* and *cxcr6^gfp/gfp^* mice. In total, 640 tracks were analyzed from four videos of *cxcr6^gfp/+^* mice, and 574 tracks were analyzed from four videos of *cxcr6^gfp/gfp^* mice. Values such as cell velocity, overall displacement, and displacement-to-path-length ratio were calculated for each track by manipulation of Volocity output in spreadsheets. To safeguard against possible flaws or biases in the Volocity software, data were analyzed according to various subsets to look for unexpected trends (such as shorter average path lengths in particular videos) that might cause the data to be biased. Furthermore, all Volocity-based conclusions, such as velocity and directedness, we confirmed by manual observation of a limited number of cells.

To calculate the visitation rate, we calculated the density of GFP^+^ cells in *cxcr6^gfp/+^* from the intravital videos used in Figure 3B and 3C (see Videos S2 and S3). Using intravital images to compare the density of GFP^+^ cells was deemed to be inaccurate because of the relatively low number of events counted and was likely to cause a bias in the choice of fields for collecting videos. Thus, the density of cells in *cxcr6^gfp/gfp^* mice was calculated from the density for *cxcr6^gfp/+^* mice and the relative frequency of cells in *cxcr6^gfp/+^* and *cxcr6^gfp/gfp^* mice as quantified by flow cytometry (see Figure 2). Using the hepatocyte nuclear exclusion of mitochondrial autofluorescence clearly visible in the higher magnification images of Figure 3A, we calculated hepatocytes to be 28 μm long along the sinusoids, at a density of 1,142/mm^2^. Utilizing the velocity data and assuming that a CD1d-reactive T cell can contact only one hepatocyte at a time, we estimated the number of hepatocyte areas visited by CD1d-reactive T cells per minute in the *cxcr6^gfp/+^* mice and in the *cxcr*6-null mice.

## Supporting Information

Video S1 NKT Cells Patrol Hepatic Sinusoids High magnification (40×) intravital video of GFP^+^ cells crawling along the hepatic sinusoids of a *cxcr6^gfp/+^* animal. Video is 300-fold compressed in time.
(6.1 MB AVI).

Video S2 NKT Cells Patrol Similarly in *cxcr6^gfp/+^* and *cxcr6^gfp/gfp^* Animals I Low magnification (10×) intravital video of *cxcr6^gfp/+^* liver showing the typical pattern of crawling observed in each genotype. Video is 300-fold compressed in time.
(9.2 MB AVI).

Video S3 NKT Cells Patrol Similarly in *cxcr6^gfp/+^* and *cxcr6^gfp/gfp^* Animals II Low magnification (10×) intravital video of *cxcr6^gfp/gfp^* liver showing the typical pattern of crawling observed in each genotype. Video is 300-fold compressed in time.
(2.3 MB AVI).

Video S4 Various Examples of Crawling Patterns of Hepatic NKT Cells Assortment of clips from various videos displaying examples of NKT cells passing each other in single sinusoids, or turning around within a single sinusoid. These examples suggest that NKT cells are not responding to spatial molecular gradients and are capable of crawling both with and against the direction of blood flow. All clips were 300-fold compressed in time.
(7.9 MB AVI).

Video S5 Real-Time Imaging of Blood Flow Low-magnification (10×) intravital video of the liver of a wild-type C57BL/6 animal during which fluorescent dextran was injected intravenously (red). Video is shown with no compression in time (i.e., at real time).
(779 KB AVI).

Video S6 NKT Cells Stop Patrolling upon Stimulation by ConA Low-magnification (10×) intravital video of the liver of a *cxcr6^gfp/+^* animal during which 250 μg of ConA was injected intravenously. Video is 300-fold compressed in time.
(4.5 MB AVI).

Video S7 NKT Cells Stop Patrolling upon Stimulation by α-GalCer Low-magnification (10×) intravital video of the liver of a *cxcr6^gfp/gfp^* animal during which 5 μg of α-GalCer was injected intravenously. Video is 300-fold compressed in time.
(7.5 MB AVI).

Video S8 Recruitment of Blood-Borne NKT Cell to Hepatic Sinusoidal Endothelium Low-magnification (10×) intravital video of the liver of a *cxcr6^gfp/gfp^* animal taken at a high frame rate (1 frame/s). The arrival, initial rolling, and subsequent attachment and crawling of a single NKT is shown.
(5.4 MB AVI).

### Accession Numbers

The LocusLink (http://www.ncbi.nlm.nih.gov/LocusLink) accession number for the CXCR6/Bonzo/STRL33 chemokine receptor is 80901and for its CXCL16 ligand is 66102.

## Abbreviations

α-GalCer: α-galactosyl ceramide
ALT: alanine aminotransferase
AST: aspartate aminotransferase
ConA: Concanavalin A
GFP: green fluorescent protein
NK: natural killer
NKT cell: natural killer T cell
PBS: phosphate-buffered saline
TCR: T-cell antigen receptor
